# Impact of telephone delivered case-management on the effectiveness of collaborative care for depression and anti-depressant use: A systematic review and meta-regression

**DOI:** 10.1371/journal.pone.0217948

**Published:** 2019-06-14

**Authors:** Joanna L. Hudson, Peter Bower, Evangelos Kontopantelis, Penny Bee, Janine Archer, Rose Clarke, Andrew S. Moriarty, David A. Richards, Simon Gilbody, Karina Lovell, Chris Dickens, Linda Gask, Waquas Waheed, Peter A. Coventry

**Affiliations:** 1 King’s College London, Department of Psychology, Institute of Psychiatry, Psychology & Neuroscience, De Crespigny Park, London, United Kingdom; 2 NIHR School for Primary Care Research, Centre for Primary Care and Centre for Health Informatics, Manchester Academic Health Science Centre, University of Manchester, Manchester, United Kingdom; 3 Division of Nursing, Midwifery & Social Work, University of Manchester, Manchester, United Kingdom; 4 School of Health and Society, University of Salford, Salford, United Kingdom; 5 Sheffield NHS Improving Access to Psychological Therapies (IAPT), St George’s Community Health Centre, Sheffield, United Kingdom; 6 Department of Health Sciences and Hull York Medical School, University of York, York, United Kingdom; 7 Institute of Health Research, University of Exeter College of Medicine and Health, University of Exeter, Exeter, United Kingdom; 8 Department for Health Sciences and Centre for Reviews and Dissemination, University of York, York, United Kingdom; University of Waterloo, CANADA

## Abstract

**Background:**

The health service delivery framework collaborative care is an effective intervention for depression. However, uncertainties remain about how to optimise its delivery at scale. Structured case management is a core component of collaborative care; its delivery via the telephone may improve access.

**Aims:**

To examine using meta-regression if telephone delivered case management diminishes the clinical effectiveness of collaborative care on depressive symptoms and anti-depressant use relative to face-to-face delivery methods.

**Methods:**

Randomised controlled trials were eligible if they included collaborative care interventions for adults with depression identified using self-report measures or diagnostic interviews and reported depression outcomes. Sociodemographics, intervention characteristics, depressive symptoms, and anti-depressant use were extracted. Random effects univariable and multivariable meta-regression analyses were used to examine the moderating effect of telephone delivered case-management on outcomes.

**Results:**

Ninety-four trials were identified comprising of 103 comparisons across 24, 132 participants with depression outcomes and 67 comparisons from 15,367 participants with anti-depressant use outcomes. Telephone delivered case management did not diminish the effects of collaborative care on depressive symptoms (β = -0.01, 95% CI -0.12 to 0.10; p = 0.86). Telephone delivered case management decreased anti-depressant medication use (relative risk 0.76, 95% CI 0.63 to 0.92; p = 0.005); this effect remained when assessed simultaneously alongside other study-level moderators of collaborative care.

**Conclusion:**

Using remote platforms such as the telephone to deliver case management may be a feasible way to implement collaborative care with no loss of effectiveness on depressive symptoms. However, adherence to anti-depressant medication may decrease when telephone case management is used.

## Introduction

Worldwide, an estimated 4.4% of the population are living with depression [1]. People with depression often do not get appropriate and timely care because health systems are not organised to deliver evidence-based treatments in an accessible format [2–4]. Collaborative care is a health service delivery framework developed to optimise depression care by using: i) multidisciplinary approaches to working with input from two or more health care professionals, ii) structured evidenced-based case management, iii) proactive and scheduled patient follow-up, and iv) enhanced inter-professional communication systems [5]. The effectiveness of collaborative care for the management of depression and anxiety in the short-term is well established [6]. However, there remains a significant translational gap between evidence of effectiveness and understanding the optimal way to implement collaborative care at scale and with reach [7]. One of the key functions of collaborative care is to enhance the way case managers can effectively work to proactively support patients to adhere to structured evidenced-based care plans. Case management may include patient contact with a health care professional to support care coordination, adherence to anti-depressant medications and/or delivery of manualised psychological interventions.

Traditionally, clinical contacts between health care professionals and patients occur via face-to-face consultations [8]. However, alternative approaches to health care delivery via telecommunication systems (e.g. telephone, conference call, web-based interfaces) are being tested; commonly referred to as telemedicine or telepsychiatry [8, 9]. Telephone delivered care has the potential to address practical, [8, 10–12] psychological [13–16] and economic barriers to accessing care [17]. Delivering depression case management by telephone within the context of collaborative care models might offer a pragmatic approach to extend the accessibility and reach of collaborative care and support its implementation at scale. However, it is not known if using the telephone to deliver case management is associated with a diminution in effectiveness of collaborative care. We therefore undertook a systematic review with meta-regression to test if the effectiveness of collaborative care is moderated by telephone delivered case management. Meta-regression is a statistical technique which performs a multiple regression of meta-analysed studies. It allows possible moderators of effect size heterogeneity between trials to be explored.

### Objectives

Our primary objective was to use meta-regression analysis to explore if trials which used telephone delivered case management moderated the effectiveness of collaborative care on depressive symptoms and use of anti-depressant medications relative to trials which used face-to-face case management delivery methods.

Our secondary objective was to explore whether including telephone delivered case management in a multivariable meta-regression model explains any additional variance in outcomes relative to other study-level moderators of collaborative care [6, 18, 19].

## Methods

This systematic review and meta-regression follows the Preferred Reporting Items for Systematic Reviews and Meta-analysis Statement guidance [20] (PRISMA; See supporting information Table A in S1 File for PRISMA checklist).

### Information sources

We originally searched the Cochrane Collaboration Depression, Anxiety and Neurosis (CCDAN) group (now Common Mental Disorders group) trial register on 9^th^ February 2012. The CCDAN trial register comprehensively indexed trials registered to MEDLINE, EMBASE, PsychINFO, CENTRAL, World Health Organisation’s trials portal, Clinicaltrials.gov, and CINAHL. The results of that search were published as a Cochrane Review [6].The search was updated using the CENTRAL database in December 2013 and incorporated in a previous meta-regression [19] and meta-analysis [21]. For this review we updated this search using the CENTRAL database in October 2016 and May 2017. This is considered a sufficient and cost-effective approach for the systematic detection of randomised controlled trials of health care interventions [22]. See Table B in S1 File for search strategy.

### Inclusion criteria

Randomised controlled trials were included if they met these criteria:

1. Recruited adults aged 18 years or over who met criteria for a primary diagnosis of depression or who had mixed anxiety and depression. Criterion thresholds were determined using either self-report questionnaires and/or diagnostic clinical interviews.
2. Used an individual or cluster randomised design, which compared collaborative care interventions in primary or community care settings with usual care or enhanced usual care.
3. Tested a type of collaborative care that included these four components [5]:
  - Multidisciplinary appraoch, defined as two or more health care professionals, of which one must include a primary care provider (e.g. family physician and/or nurse practitioner).
  - Structured evidence-based case management plan delivered by a health care professional/case manager who is not the patient’s primary care provider. Case management plans could include pharmacotherapy and/or psychotherapy.
  - Scheduled and proactive patient follow-up consisting of one or more planned sessions.
  - Enhanced inter-professional communication/support, for example: team meetings, supervision from a senior health care professional/mental health specialist.
4. Measured change in depressive symptoms using self-report measures or diagnostic clinical interviews. Binary self-report depression outcomes may have included either remission or reduction in depression symptoms according to a priori defined threshold (e.g. ≥50%).

### Study selection

Eligible studies were identified for inclusion from our existing meta-regression of 74 collaborative care randomised controlled trials for depression [19]. In addition, three authors (JH, PC, RC) screened potentially eligible studies identified from the CENTRAL search updates against the above inclusion criteria.

### Data extraction

Data extraction focussed on: i) characteristics of included studies, ii) characteristics of collaborative care interventions, iii) depressive symptoms and antidepressant medication use, and iv) categorical coding of study level moderators of collaborative care.

#### Characteristics of included studies

To summarise trial characteristics the following variables were extracted: sample size, sociodemographic data, method used to diagnose depression, and baseline depression severity.

#### Characteristics of collaborative care interventions

Consistent with Gunn et al’s [5] conceptualisation of collaborative care, we extracted the following descriptive data: i) type of health care professionals responsible for case management, ii) type of evidence-based case management plan implemented (e.g. pharmacotherapy, psychotherapy or both), iii) number of planned follow-up case-management sessions (during 6 month period), and iv) methods used to enhance inter-professional communication.

#### Outcome extraction

The primary outcome was reduction in depressive symptoms. We extracted continuous or dichotomous depression outcomes for follow-up data that was closest in time to six months. If studies included two active comparator trial arms relative to the control group, we halved sample sizes to prevent double counting. The *metaeff* stata command [23] was used to translate dichotomous outcomes into standardised mean differences. This allowed the inclusion of both continuous and dichotomous outcomes in the same analysis.

Anti-depressant use is an important process variable. Adherence to correctly prescribed medication will improve depression outcomes. We extracted antidepressant use as a dichotomous outcome and translated the data into relative risk ratios with log-transformations among any studies that reported this process variable [24]. Data were extracted from studies that reported either: the proportion of patients who were using anti-depressants or the proportions of patients meeting a priori defined cut-offs for appropriate levels of medication use according to self-report measures or clinical guidelines.

If trials used a cluster randomisation process, then we used standard approaches to implement the “effective sample size” procedure [25]. We used an empirically-derived intraclass correlation coefficient (ICC) of 0.02 [26].

#### Categorical coding of case-management delivery method and study level moderators of collaborative care

To examine if telephone delivered case management moderated depressive symptom outcomes and anti-depressant use a binary code (e.g. telephone versus face-to-face) was applied to each trial. We conceptualised telephone delivered case management as planned treatment sessions delivered by the case manager over the telephone. The case manager’s role may have involved supporting adherence to evidence-based pharmacotherapy and/or psychotherapy treatments. If planned case management sessions included a mix of telephone and face-to-face delivered sessions, then ≥50% of the planned case management sessions had to be delivered via the telephone for a study to be coded as telephone delivered case management intervention.

In addition, the following study-level moderators were categorically coded: i) participant recruitment method (e.g. systematic screening methods using either diagnostic clinical interviews or self-report depression measures versus referral by clinicians), ii) intervention content delivered during case management sessions (e.g. pharmacotherapy only versus psychotherapy and/or pharmacotherapy), and iii) supervision frequency (e.g. scheduled supervision versus ad hoc). These three study-level moderators were selected for extraction because our previous meta-regression analysis identified them as potentially salient moderators of depression outcomes and/or anti-depressant use [19]. This approach made it possible to test if telephone delivered case management adds any additional explanatory power over and above our previously tested multivariable explanatory models of depression outcomes and anti-depressant use.

### Analysis

Before performing meta-regression analyses to compare the effect of collaborative care trials which delivered case management via the telephone with trials that used face-to-face case management delivery methods we first performed a meta-analysis to standardise depressive symptom and anti-depressant use outcomes. To achieve this the Stata (StataCorp LLC; Version 15 for windows) command *metaan [27]* was used to generate standardised mean difference effect size estimates for depressive symptoms (meta-analysis model one) and relative risk effect size estimates for anti-depressant use outcomes (meta-analysis model two). In both cases, a DerSimonian-Laird [28] random-effects inverse variance model was used to better account for heterogeneity. The I^2^ estimate was used to estimate the degree of heterogeneity across included studies; it provides a percentage estimate of between study variability [29]. We also report the 95% confidence intervals for I^2^.

Four meta-regression analyses were then performed usingthe *metareg* [30] command. Univariable meta-regression analyses were performed to test our primary objective. We examined if telephone delivered case management moderated the effect of collaborative care on depressive symptoms (univariable meta-regression model one) and anti-depressant use (univariable meta-regression model two). The *metareg* command was also used to test our secondary objective. Using multivariable meta-regression analyses, we examined if telephone delivered case management added explanatory effects on outcomes relative to our previously tested study-level moderators of collaborative care outcomes [19]. We simultaneously entered the following study-level explanatory variables into a meta-regression model with depressive symptoms as the outcome variable: telephone delivered case management, recruitment method, intervention content, and supervision frequency (multivariable meta-regression model three). These three variables were selected for inclusion in the model to replicate our previously tested study-level moderators [19]. Likewise, when anti-depressant use was the outcome variable we simultaneously entered: telephone delivered case management alongside recruitment method (multivariable meta-regression model four). Recruitment method was identified for inclusion in the model to replicate our previous multivariable study-level meta-regression findings for anti-depressant use [19].

### Risk of bias

Study sample size was used as a proxy indicator for publication bias by exploring the relationship between study effect size and sample size [31]. We quantified the effect of risk of within study bias associated with allocation concealment which was coded as a binary variable [32].

## Results

### Characteristics of included studies

**Fig 1:**
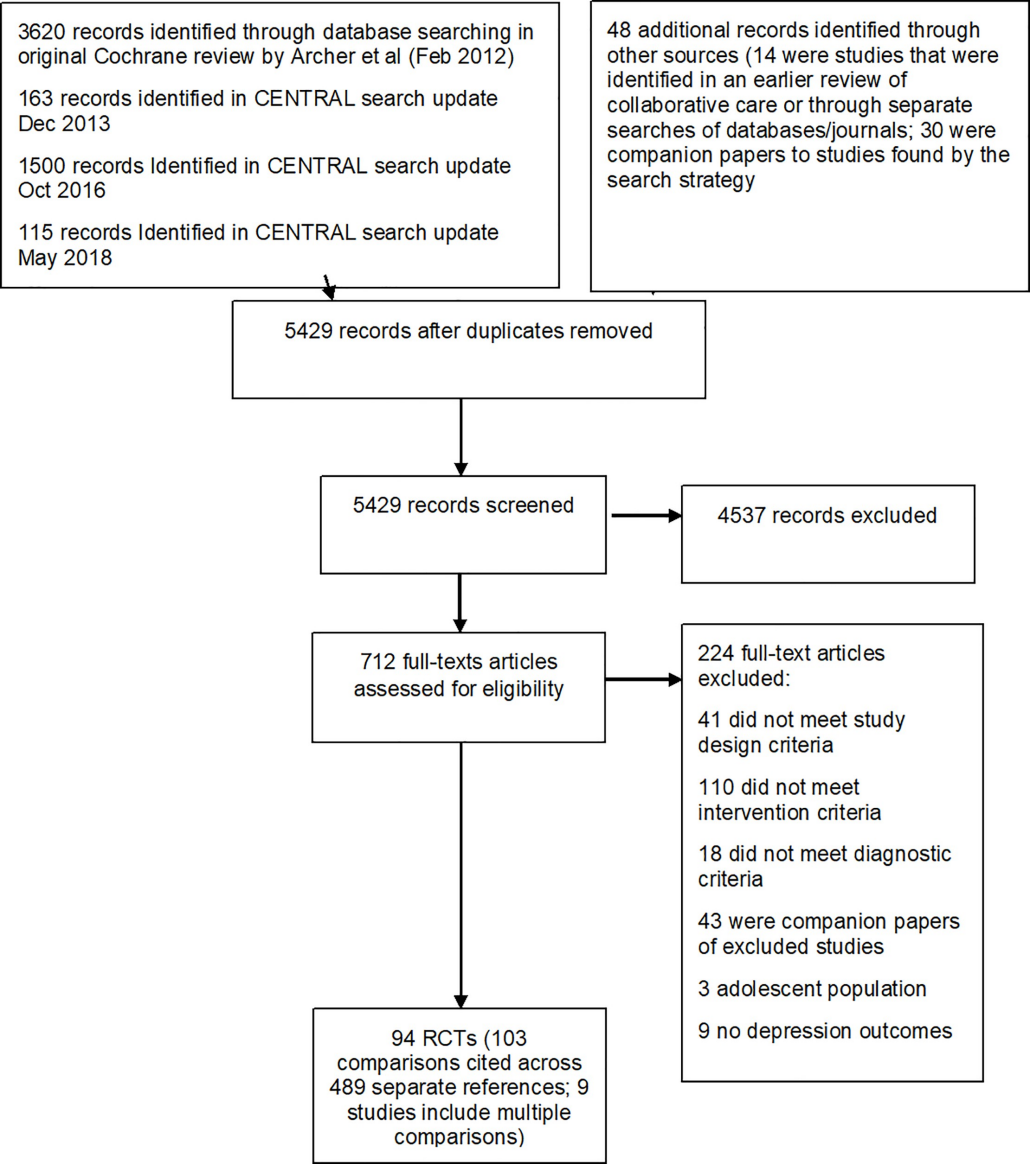
PRISMA flow diagram.

Ninety-four trials were identified that included 103 comparisons from 24, 132 participants with depression outcomes and 66 comparisons from 15, 367 participants with anti-depressant use outcomes; see Fig 1 for PRISMA flow diagram. This represents a 27% increase on the number of trials included in our previous systematic review with meta-regression [19]. Collaborative care case management was delivered using the telephone in 42% (n = 43) of the included trial comparisons. Across the 103 comparisons, 53 comparator groups used a mental health care professional to deliver their case management. Most of the intervention comparators opted for scheduled supervision (62%). A summary of the collaborative care characteristics for each trial is provided in Table C S1 File. A third of the comparator trials were conducted in the US, whilst only 3% (n = 3) were conducted in low and middle-income countries. Characteristics of included studies are described in Table D S1 File. A reference list of all included studies is available in Reference List AS1 File.

#### Meta-analysis model one: Depressive symptoms

Meta-analysis findings showed that collaborative care was associated with greater improvements in depressive symptoms when compared with usual care (standardised mean difference, SMD −0.30, 95% CI −0.36 to −0.25; p<0.001; I^2^ = 73.1%, 95% CI 67.7% to 78.0%, k = 103).

#### Meta-analysis model two: Anti-depressant use

Meta-analysis findings showed that collaborative care was associated with greater use of anti-depressant medications when compared with usual care (relative risk, RR 1.48, 95% CI 1.37 to 1.59; p<0.001; I^2^ = 80.1%, 95% CI 75.2% to 84.1%, k = 66).

#### Univariable meta-regression model one: The moderating effect of telephone delivered case management on depressive symptoms

**Fig 2:**
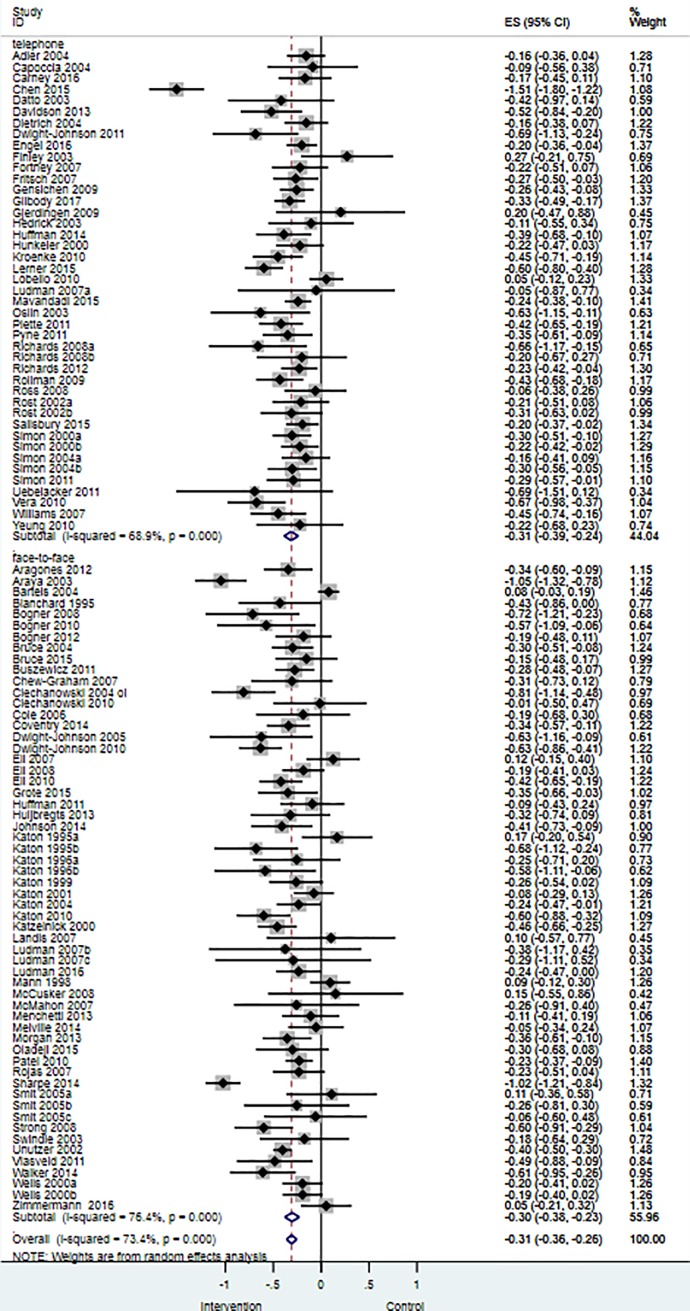
Effects of collaborative care on depression outcomes for studies that used telephone delivered case management versus face-to-face delivered case management. Intervention = Collaborative care; Control = Usual care or enhanced usual care.

Univariable meta-regression analyses showed that telephone delivered case management did not have a statistically significant moderating effect on depression outcomes (study level β = -0.01, 95% CI -0.12 to 0.10; p = 0.86; I^2^ = 73.7%, k = 101). Comparable depressive symptom effect size estimates were observed for trials which used telephone case management delivery methods relative to face-to-face delivery as are summarised in Fig 2.

#### Univariable meta-regression model two: The moderating effect of telephone delivered case management on anti-depressant use

**Fig 3:**
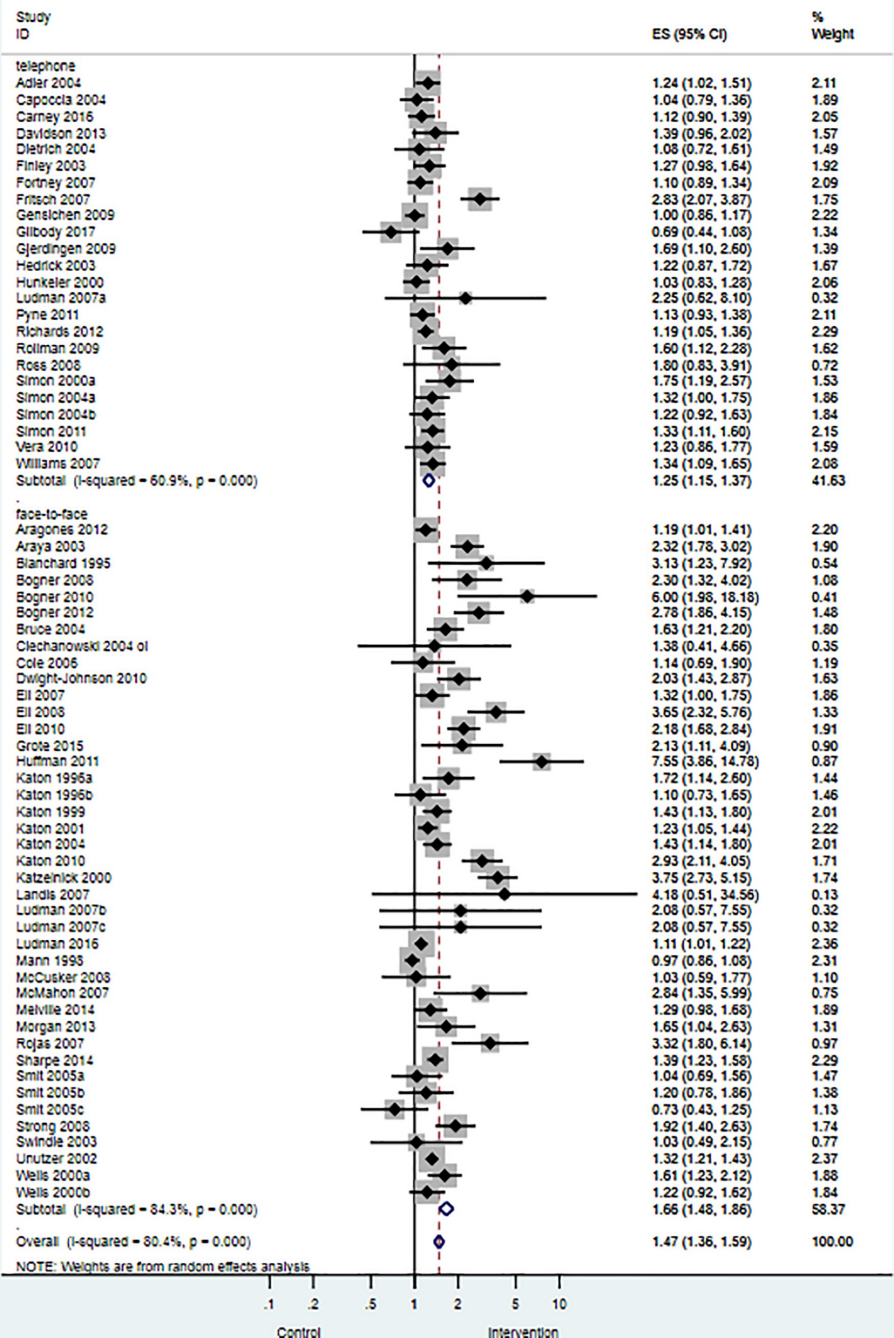
Effects of collaborative care on anti-depressant use for studies that used telephone delivered case management versus face-to-face delivered case management. Intervention = Collaborative care; Control = Usual care or enhanced usual care.

Univariable meta-regression analyses showed that telephone delivered case management had a statistically significant moderating effect on anti-depressant use (RR = 0.76, 95% CI 0.63 to 0.92; p = 0.005; I^2^ = 79.6%, k = 66). Studies that delivered case management via the telephone reported lower use of anti-depressant medications. Fig 3 summarises the effect size differences for anti-depressant use across telephone delivered case management studies versus studies that used face-to-face delivered case management.

#### Multivariable meta-regression model three: Testing telephone delivered case management relative to other moderators of depressive symptoms in collaborative care trials

Multivariable meta-regression analyses showed that telephone delivered case management did not have a statistically significant explanatory effect on depressive symptoms when entered simultaneously alongside the following explanatory variables: study recruitment method, intervention content, and supervision frequency. Only scheduled supervision frequency (relative to ad hoc supervision frequency; study level ß = -0.16, 95% CI -0.27 to -0.04, p = 0.008, k = 101) had a statistically significant effect on depressive symptoms. Studies that had regular scheduled supervision reported greater improvements in depression outcomes relative to studies which used ad-hoc supervision structures. Table 1 summarises the findings of multivariable meta-regression model three.

**Table 1:** Multivariable meta-regression model three–outcome depressive symptoms.

Explanatory Variables		Regression Coefficient (95% CI)	SE	P
Case management delivery method	Telephone (vs face-to-face)	0.02 (-0.09 to 0.14)	.06	.681
Recruitment method	Systematic (vs GP referral)	-0.11 (-0.25 to 0.03)	.06	.106
Intervention content	Psychotherapy or both (vs medication only)	-0.04 (-0.16 to 0.07)	.06	.451
Supervision frequency	Scheduled (vs ad hoc)	-0.16 (-0.27 to -0.04)	.06	.008
	Not applicable (vs ad hoc)	0.07 (-0.21 to 0.34)	.14	.579

101 comparisons, I^2^ = 67.93

Key: CI–Confidence interval

#### Multivariable meta-regression model four: Testing telephone delivered case management relative to other moderators of anti-depressant use in collaborative care trials

Multivariable meta-regression analyses showed that case management delivery method remained as a statistically significant explanatory variable when entered simultaneously alongside study recruitment method. Trials which used telephone-delivered case management relative to face-to-face case management were statistically less likely to use anti-depressant medication (study level RR = 0.76, 95% CI 0.64 to 0.91, p = 0.002, k = 66). Likewise, recruitment into the trial using systematic methods of identification relative to opportunistic identification of depression improved anti-depressant medication use (study level RR = 1.45, 95% CI 1.16 to 1.80, p = 0.001, k = 66). Table 2 summarises the findings from the multivariable meta-regression model four.

**Table 2:** Multivariable meta-regression model four–outcome anti-depressant medication use.

Explanatory Variables		Relative risk (95% CI)	SE	P
Telephone support	Telephone (vs Face-to-face)	0.76 (0.64 to 0.91)	.07	.002
Recruitment method	Systematic (vs GP referral)	1.45 (1.16 to 1.80)	.16	.001

66 comparisons, I^2^ = 77.1

Key: CI = Confidence interval

#### Publication bias

We statistically tested for publication bias by exploring the relationship between effect size and sample size. These findings were statistically non-significant for depressive symptoms and anti-depressant use. These outcomes reduce the likelihood that our findings are vulnerable to publication bias.

#### Risk of within study bias

There was no statistically significant effect of allocation concealment on depressive symptoms or anti-depressant use, decreasing the likelihood that our findings are impacted upon by trial quality.

## Discussion

We conducted a meta-regression analysis to examine whether trials that used telephone delivered case management methods diminished the effect of collaborative care on depressive symptoms relative to collaborative care trials that tested face-to-face case management delivery methods. We found no evidence to support this, therefore suggesting that telephone and face-to-face delivered case management delivery methods have equivalent effects on depressive symptoms when implemented as part of a collaborative care intervention. Consistent with our previous meta-regression [19], use of scheduled and regular supervision from a senior clinician were the only study-level moderators which bolstered the effects of collaborative care on depressive symptoms. Processes of care, specifically use of anti-depressant medication, were improved in trials that used face-to-face delivered case management relative to telephone delivered case management. Case-management delivery method remained a statistically significant explanatory variable when entered simultaneously alongside other hypothesised study-level moderators of anti-depressant use in collaborative care contexts. It is important to highlight that these findings are based on meta-regression observational analyses only and the implications of this are discussed below.

### Strengths and limitations

This is the first systematic review with meta-regression to assess the impact of telephone delivered case management as part of a collaborative care intervention on depressive symptoms and processes of care. In addressing this novel research question we also updated the findings of a previous Cochrane review [6]. This provided an opportunity to replicate previous multivariable meta-regression analyses using a dataset that included 21 more trials [19]. Using this enlarged dataset increased statistical power [33] and improved the reliability of our effect size estimates which rely on asymptomatic sampling methods [34]. However, large amounts of statistical heterogeneity remain unexplained in both multivariable meta-regression models. The capability of meta-regression analyses to explain the impact of clinical and methodological heterogeneity on trial outcomes is reliant on the comprehensive reporting of these characteristics. CONSORT guidelines have improved methodological reporting and assessment of trial quality [35]. Indeed, we explored the effect of trial quality using allocation concealment as a hypothesised moderator of outcome. However, the reporting of intervention characteristics, specifically their “active ingredients” and planned vs actual processes of intervention delivery (e.g. number and intensity of treatment sessions), remain poorly reported. The template for intervention description and replication (TIDieR) checklist provides a framework for reliably reporting this information [36]. Using TIDieR within trial reports will enhance the capabilities of future meta-regression analyses to explore these factors with precision and statistical power. In addition, trials that seek to elaborate on how collaborative care is implemented need to go a step further and consider the potential mechanisms of action through which intervention effects are produced [37–39]. This will allow intervention developers to identify what aspects of their evidenced-based treatment protocols can remain the same (e.g. when mechanisms of action respond to clinical intervention as hypothesised) versus which aspects of the treatment protocols need updating when hypothesised mechanisms of change do not respond to clinical intervention method(s) used. However, our systematic review with meta-regression found that only two thirds of the included studies measured change in anti-depressant medication use which is an important process variable. Improved reporting of patient characteristics and treatment components as well as measuring hypothesised mechanisms of change, will more likely lead to a better understanding of how to maximise the benefits of collaborative care and tailor delivery to meet the needs of patients [39].

Nonetheless, meta-regression remains reliant on aggregate level data extracted from across trials. Observed relationships between identified study-level moderators may be confounded by individual patient-level characteristics (e.g. gender, patient preference for telephone case management) or indeed other between-trial characteristics (e.g. city versus rural settings) [40]. The potential for between-trial confounding was offset by our use of multivariable meta-regression analyses, but unmeasured confounding at the individual and study level may still have occurred [41]. To truly establish the impact of telephone delivered case management on outcomes comparative effectiveness randomised controlled trials of collaborative care which directly compare telephone with face-to-face delivery methods are needed. We are aware of only one such depression trial to date which was previously not included in our effectiveness reviews because the comparator was not usual care [42]. The study found that telephone case management had larger effects on depression outcomes when compared with face-to-face delivery methods when offered in a rural setting in the US.

### Interpretation of findings and implications for research, practice, and policy

The UK Five Year Forward View for Mental Health emphasises the need to drive and scale improvements in mental health services with priorities focused on access, quality and integrated care [43]. Within this policy context there is scope to prioritise the implementation of services that overcome practical and psychological barriers to accessing depression treatments [10–16]. Telephone delivered cognitive-behavioural therapy (CBT) is associated with equivalent improvements in depression outcomes and lower attrition when compared with face-to-face delivery methods, but possibly at the expense of maintaining gains in depression outcomes when treatment ends [44]. Therefore, telephone delivered case management, implemented as part of a collaborative care framework, may improve depression outcomes for up to six months and possibly beyond [45] whilst retaining equivalent effects as face-to-face case management. In this sense, telephone delivered case management, as part of collaborative care, is a promising intervention to translate into practice the goals of the Five Year Forward View [43] by providing a platform to implement high quality mental health care at scale and with reach.

When used as part of collaborative care, telephone delivered case management is an acceptable and feasible way to increase access to mental health care among hard to reach communities, including low income and ethnic minority groups who live in high income countries [46]. This may partly owe to the fact that telephone contacts can reduce stigma associated with attendance with mental health clinics [47]. Additionally, telemedicine offers greater choice to people who prioritise access and availability of services over the co-location of practitioner and patient [14]. The telephone is the most ubiquitous communication technology. Advances in connectivity and smart phone technology have led to the rapid spread of mobile phone use. Globally the number of mobile technology users is expected to surpass 5 billion by 2019 with over 70% of users concentrated in low and middle income countries (LMIC) [48]. In LMIC countries geographic barriers to accessing mental health care might prevail making telephone delivered case management the optimal way to deliver structured care plans as part of collaborative care.

However, prioritising the implementation of collaborative care on the grounds of access and reach should not compromise patient centred care that meets patients’ expectations and preferences. The relationship between patient and practitioner (the therapeutic alliance) is an important mechanism for improving outcomes in psychological therapy [49]. For some patients, telephone case management as part of collaborative care may be perceived as too impersonal and anonymous, possibly reducing opportunities for generating a therapeutic alliance [50]. However, studies that have quantitatively assessed this variable report no significant differences in patients’ ratings of alliance between telephone and face-to-face interventions [51, 52]. There is then scope to better understand and model which patients might prefer or benefit from telephone case management using rich datasets collected as part of individual patient data meta-analyses of the effectiveness of collaborative care [53]. In addition, understanding how to optimise the delivery of telephone delivered case management by considering, for example, dose-response relationships using the latest statistical modelling techniques is needed [54]. However, to robustly study these factors they must be appropriately considered at the trial design phase [39].

The observation that mode of delivery does not impact on depression outcomes but does reduce adherence to anti-depressant medication is notable. Limited research in non-mental healthcare settings suggests that patients may possess clear beliefs about how they value the role of communication technologies in their care [55]. While telemedicine is perceived as appropriate for routine consultations, richer face-to-face exchanges have traditionally been associated with the receipt of more complex treatments. Thus, users of health services are likely to hold a variety of beliefs regarding remotely-delivered interventions depending upon the context in which their interactions occur. When telephone interventions are delivered as part of a collaborative care it is possible that they are predominantly seen by patients and providers as a means to coordinate care and/or deliver psychological therapies rather than monitor medication use and adherence. However, this proposition needs to be empirically validated using qualitative methods to generate greater understanding about patient and provider beliefs about the merit and value of using the telephone as part of collaborative care. To achieve these aims, exploring patients’ perceived acceptability and utility of treatment interventions and their mode of delivery may help clinicians identify potential barriers to uptake and treatment fidelity and address these directly. Theoretical frameworks on acceptability and utility of treatment interventions may help to guide clinicians’ lines of Socratic questioning [56].

### Conclusion

This meta-regression analysis suggests that the use of the telephone to deliver case management does not reduce the effectiveness of collaborative care on depression outcomes. The results for depression outcomes were no different in trials of collaborative care that used telephone case management than trials that used face-to-face case management. However, trials that used telephone delivered case-management reported reduced adherence to anti-depressant medication highlighting the need to consider how the mode of delivery may shape patient and health care professionals understanding about the purpose of the case management. Embedding telemedicine within collaborative care frameworks has the potential to improve access and reach of high quality mental health care globally.

## Supporting information

S1 File (DOCX)

S1 Data Data file with extracted outcomes and covariates. (XLSX)
